# Towards Adaptive Grids for Atmospheric Boundary-Layer Simulations

**DOI:** 10.1007/s10546-018-0335-9

**Published:** 2018-02-14

**Authors:** J. Antoon van Hooft, Stéphane Popinet, Chiel C. van Heerwaarden, Steven J. A. van der Linden, Stephan R. de Roode, Bas J. H. van de Wiel

**Affiliations:** 1 Department of Geoscience and Remote Sensing, Delft University of Technology, Delft, The Netherlands; 2 Sorbonne Université, Centre National de la Recherche Scientifique, UMR 7190, Institut Jean Le Rond d’Alembert, F-75005 Paris, France; 3 Department of Environmental Sciences, Wageningen University and Research, Wageningen, The Netherlands

**Keywords:** Adaptive mesh refinement, Atmospheric boundary layer, Direct numerical simulations, Large-eddy simulations, Turbulence

## Abstract

We present a proof-of-concept for the adaptive mesh refinement method applied to atmospheric boundary-layer simulations. Such a method may form an attractive alternative to static grids for studies on atmospheric flows that have a high degree of scale separation in space and/or time. Examples include the diurnal cycle and a convective boundary layer capped by a strong inversion. For such cases, large-eddy simulations using regular grids often have to rely on a subgrid-scale closure for the most challenging regions in the spatial and/or temporal domain. Here we analyze a flow configuration that describes the growth and subsequent decay of a convective boundary layer using direct numerical simulation (DNS). We validate the obtained results and benchmark the performance of the adaptive solver against two runs using fixed regular grids. It appears that the adaptive-mesh algorithm is able to coarsen and refine the grid dynamically whilst maintaining an accurate solution. In particular, during the initial growth of the convective boundary layer a high resolution is required compared to the subsequent stage of decaying turbulence. More specifically, the number of grid cells varies by two orders of magnitude over the course of the simulation. For this specific DNS case, the adaptive solver was not yet more efficient than the more traditional solver that is dedicated to these types of flows. However, the overall analysis shows that the method has a clear potential for numerical investigations of the most challenging atmospheric cases.

## Introduction

The aim of the present study is to introduce adaptive mesh refinement (AMR) as an efficient tool for numerical investigations of the atmospheric boundary layer (ABL) using turbulence resolving methods. This refers typically to models that rely on direct numerical simulation (DNS) or large-eddy simulation (LES) techniques. In general, AMR solvers aim to distribute the available computational resources efficiently over a domain by dynamically refining and coarsening the computational grid in space and time. AMR techniques have successfully been employed in studies concerning flows with a high degree of scale separation throughout the spatial and/or temporal domain. Such studies concern a wide range of topics, e.g. cosmological hydrodynamics (Teyssier 2002), electro hydrodynamics (López-Herrera et al. 2011), multiphase flows (Fuster et al. 2009), flows in complex geometries (Popinet 2003) and turbulence simulations (Schneider and Vasilyev 2010). However, to our knowledge, the potential of this technique has not yet been explored for ABL research, and here we aim to do so through an investigation of the consecutive growth and decay of a convective boundary-layer (CBL) system. The flow configuration is modelled after Heerwaarden and Mellado 2016 who performed an in-depth study of this case using a regular grid configuration. As such, the AMR method is tested and benchmarked.

Several methods that meet a varying resolution requirement throughout the spatial domain have already been successfully applied in studies on ABL turbulence. For example, stretching and squeezing of grids (see e.g. Heus et al. 2010; Heerwaarden and Mellado 2016; Roode et al. 2016), nested grids (see e.g. Sullivan et al. 1996, 1998; Moeng et al. 2007; Mirocha et al. 2013; Muñoz-Esparza et al. 2014) and the usage of unstructured anisotropic grids. However, the mesh is always kept fixed during the simulation, whereas dynamical changes in the ABL call for variation of resolution in time. Furthermore, the aforementioned methods of refinement need to be predefined. Consequently, detailed a priori knowledge is needed on the varying resolution requirement throughout the spatial domain. Apart from tailored and well-known cases, this knowledge is usually not available beforehand; therefore, we identify three favourable characteristics of an AMR approach for ABL studies. First, the resolution can vary throughout the spatial domain. Second, the grid can vary in time such that temporal variation in the local resolution requirement can be met. Third, the grid is generated adaptively based on the evolution of the numerical solution itself, relaxing the requirement of detailed a priori knowledge on the resolution requirement.

**Fig. 1:**
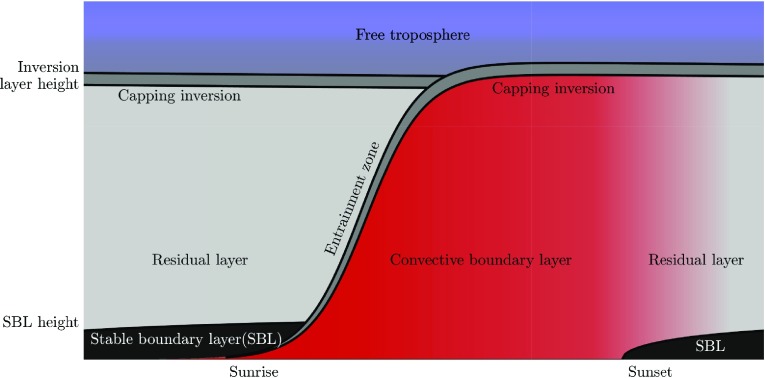
Sketch of a prototypical diurnal cycle evolution. Adapted from Stull (1988)

To illustrate our philosophy, we briefly discuss a textbook example of the evolution of the ABL during a diurnal cycle (after Stull 1988). Figure 1 depicts a typical evolution of the ABL during a diurnal cycle. Around sunrise the solar irradiation of the Earth’s surface causes a thermal instability that results in the rapid growth of a CBL. The typical size of the largest thermal plumes scales with the boundary-layer height and hence there is a temporal dependency on the resolution requirement to resolve these turbulent structures. The growth of the ABL slows down when the rising thermals reach the inversion layer, which effectively caps turbulent structures at the top of the CBL. The dynamics within an inversion layer are of pivotal importance for the evolution of the CBL (Garcia and Mellado 2014). Apart from the effective ‘lid’ on the boundary layer, entrainment processes occur here and the formation of stratocumulus clouds is promoted by the large jump in temperature with height. Due to the presence of strong stable stratification, turbulent length scales are suppressed (Lozar and Mellado 2015), and in order to resolve the most prominent turbulent structures here, a much higher resolution is necessary compared to the bulk of the CBL (Sullivan and Patton 2011; Lozar and Mellado 2015). Applying such high resolution everywhere in the domain is not feasible given the current status of computational resources, and might not be feasible in coming years (Bou-Zeid 2015). For this reason, many LES studies have to rely on their subgrid-scale (SGS) parametrizations within the region of the inversion layer, partially negating the purpose of a turbulence resolving study. Furthermore, the exact height and strength of the inversion layer are not always known a priori (except in cases that have been studied before). Fixed nested grids (Sullivan et al. 1998) are thus not always flexible enough to capture the dynamics properly. On the other hand, practically speaking, it should be noted that LES results between various studies often tend to converge, signifying that SGS models have appreciable skill in describing certain characteristics of the inversion layer (see e.g Nieuwstadt et al. 1993; Siebesma et al. 2003).

At the approach of sunset, thermal plumes gradually decay into so-called residual turbulence, and due to the radiative cooling of the Earth’s surface, stable stratification sets in and turbulence is now driven by wind shear only. The stable boundary layer (SBL) is typically much shallower than the CBL and, furthermore, the length scales of the turbulent structures that account for the mixing of heat and momentum within this layer are only a fraction of the size of those associated with daytime convective turbulence (Basu et al. 2008). Additionally, Ansorge and Mellado (2016) argue that the resolution requirement for their simulations of the intermittently turbulent SBL is dictated by localized dissipative flow structures that only encompass a fraction of the computational domain.

Rather than capturing the cyclic behaviour of the atmosphere as depicted in Fig. 1, the contrast between daytime and night-time turbulence has resulted in many numerical studies focusing only on either convective or stable conditions. The studies that do simulate a diurnal cycle typically struggle to resolve turbulence during the night (Kumar et al. 2006; Basu et al. 2008; Abkar et al. 2015). Furthermore, the transition period itself (i.e. around sunset) would benefit from high fidelity numerical studies (Lothon et al. 2014). In summary: the example shows that the intrinsic dynamic character of the ABL calls for flexible techniques such as an AMR appoach in addition to existing techniques that have successfully been applied to studies on idealized, steady cases.

Apart from our long-term prospects, we focus here on a case corresponding to the red and grey sections in Fig. 1. This choice is motivated by the fact that as a first step, we would like to present a proof-of-concept of the AMR approach before we redirect our attention towards more challenging cases. Therefore, we present results obtained with DNS, for which all turbulent structures are resolved explicitly down to the small-scale Kolmogorov length (i.e. the viscous length scale) according to the Navier–Stokes equations, without any closure for turbulence. Compared to, for example, LES, the results obtained with DNS should be independent of the numerical formulations or choice of any SGS model, whereas with LES this is a topic of discussion (Bretherton et al. 1999; Siebesma et al. 2003; Fedorovich et al. 2004; Beare et al. 2006; Roode et al. 2017). However, as shown in Sect. 4, the concept of the AMR approach can be easily extended to LES. Since this technique is a popular choice for studies on the ABL, we also briefly discuss results obtained with the AMR technique using a LES formulation.

We realize that it is impractical to address all questions regarding the AMR technique in relation to ABL simulations. For example, here we focus on a single case whereas we will argue that the performance of an AMR solver varies depending on the particular case specifications (see Appendix 1). Furthermore, we choose a numerical solver called Basilisk (http://basilisk.fr) for the adaptive-grid runs and do not assess alternatives.

The paper is organized as follows; in Sect. 2.1 the details of the adaptive-grid solver are described, focusing on the AMR algorithm, and in addition, Sect. 2.2 provides an example analysis of how the algorithm assesses a turbulent signal and adapts the grid accordingly. In Sect. 2.3 the case and the numerical set-up of the different runs are specified. Section 3 presents the obtained results including a performance assessment, while in Sect. 4 we provide an outlook on future plans. We finish with a conclusion combined with a discussion in Sect. 5. Additionally, using a simple flow set-up, Appendix 1 illustrates an important advantage the AMR technique has over a fixed equidistant-grid approach.

## Methods

### Basilisk and the Grid Adaptation Algorithm

The AMR runs are performed with the partial-differential-equation solver called Basilisk, a code that contains a second-order accurate finite-volume solver for the Navier–Stokes equations. For a detailed description of the numerical formulations, see Popinet (2003, 2009), Lagrée et al. (2011), and references therein.

**Fig. 2:**
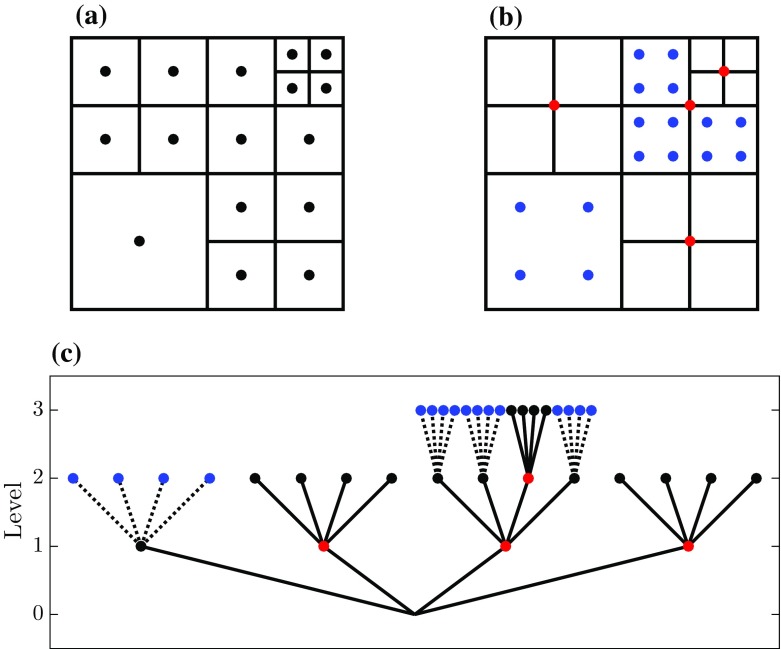
Example of a tree-grid structure. The top row presents the spatial structure of the grid cells with varying levels of refinement (**a**) and the locations of two types of ghost points whose field values are defined by the downsampling (red dots) and upsampling (blue dots) operations (**b**, see text). The plot on the bottom row presents a corresponding tree representation of the various grid cells and ghost points at different levels (**c**)

**Fig. 3:**
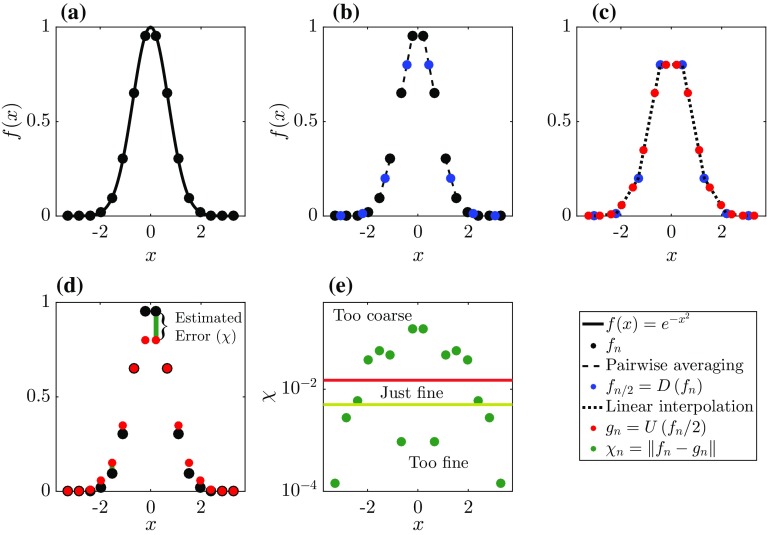
A one-dimensional, visual representation of how the adaptation algorithm assesses the discretization of a curved field *f*(*x*): **a** A coarser level estimate of the discretized solution is obtained using the downsampling operation. **b** Using these coarse level values, the original discretized solution can be estimated using the upsampling operation. **c** The difference between the estimated and original values is interpreted as an error estimator ($\chi$) and can be compared against fixed thresholds (e.g. $\zeta$). **d**, **e** If the refinement criterion is exceeded, new cells at one level higher are initialized locally by applying a linear interpolation technique using the initial cell values. Alternatively, if the estimated error is smaller than the coarsening criterion for multiple cells, these cells can be merged if that criterion does not violate the general grid-structure requirements (see text and Fig. 2)

In order to facilitate local adaptive refinement and coarsening whilst maintaining a Cartesian grid-structure, a so-called tree-based grid is used. To illustrate this mesh structure, Fig. 2 shows the two-dimensional (2D) variant of a tree-based grid (i.e. a quadtree), whose structure introduces a hierarchy between cells at integer levels of refinement. The resolution between the levels of refinement differs by a factor of two and the Basilisk solver allows neighbouring cells to vary up to one level. The formulations of numerical methods (e.g. evaluating spatial derivatives) on equidistant Cartesian grids are relatively straightforward compared to their uneven grid counterparts. Therefore, ghost points are defined, enabling simple Cartesian stencil operations for the cells in the vicinity of a resolution boundary. These points act as virtual cells and are located such that all cells have neighbours that are defined at the same level of refinement, see Fig. 2b. The field values on these ghost cells are defined with interpolation techniques using the original field values.

The tree grid facilitates an efficient and convenient structure to perform a multiresolution analysis of a discretized field. During the simulation, such an analysis is used to determine which grid cells require refinement and where in the domain cells can be coarsened. This procedure is discussed next. Consider a 1D signal (*f*) discretized with an even number (*n*) of elements $f_n$, where individual entries of $f_n$ are indexed with *i* such that $f^i_n$ represents the *i*th entry of $f_n$. First, we define a downsampling operation (*D*) that approximates $f_n$ on a coarser level grid with *n* / 2 elements,

1 $$\begin{aligned} f_{n/2}=D(f_n). \end{aligned}$$

Second, we define an upsampling operator (*U*) that samples $f_{n/2}$ to a signal that is defined with the same element entries as the original signal $f_n$,

2 $$\begin{aligned} g_{n}=U(f_{n/2}), \end{aligned}$$

noting that in general $f_n \ne g_n$, and the absolute difference $\chi$, defined as,

3 $$\begin{aligned} \chi ^i_{n}=\Vert f^i_n-g^i_n\Vert , \end{aligned}$$

can be interpreted as an estimation of the discretization error. The downsampling operation in the Basilisk solver is defined as local volume averaging of the signal to obtain a value for a corresponding coarser-level grid cell (see Fig. 3a). This formulation is exact since in a finite-volume formulation, the grid cell values represent volume-averaged quantities. To be in line with the second-order accuracy of the solver, the upsampling operation is chosen to be second-order accurate as well, and entails performing a linear interpolation between the grid points of the coarse level solution (see Fig. 3b). Once these two operations have been applied to the discretized signal, it is possible to evaluate $\chi ^i_n$ for each of the grid cells. Given an error threshold $\zeta$, the following assessment with regards to the grid-cell resolution can be made,

4 $$\begin{aligned} \text {the }i\text {-th grid cell is } {\left\{ \begin{array}{ll} \text{ too } \text{ coarse. } &{} \chi ^i_n > \zeta ,\\ \text{ too } \text{ fine. } &{} \chi ^i_n < \frac{2\zeta }{3},\\ \text{ just } \text{ fine. }&{}\text{ Otherwise }. \end{array}\right. } \end{aligned}$$

The threshold on the estimated error for refinement $\zeta$ is called the refinement criterion, with $\zeta$ having the same physical units as *f*. Note that the described method is formally linked to wavelet thresholding that has already been employed for fluid dynamical simulations (Schneider and Vasilyev 2010). The grid can be refined and coarsened according to Eq. 4, and field values for the new refined and coarsened cells can be defined using an identical formulation as is used for the *U* and *D* operator, respectively. However, the Basilisk solver allows the formulations for upsampling and downsampling during the grid-resolution assessment and the actual refinement and coarsening of cells to differ.

**Fig. 4:**
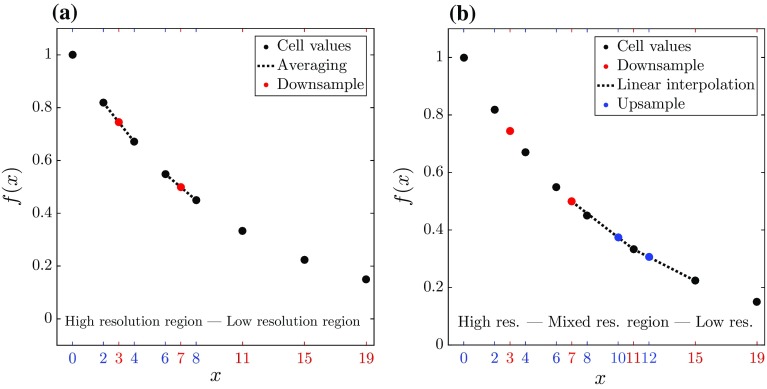
Example of the treatment of a resolution boundary in a one-dimensional scenario. First, the high-level region near the resolution boundary is downsampled to obtain values for the coarse-level ghost points in red (**a**). Second, linear interpolation of the coarse level solution is used to define the field values of high-level ghost points in blue (**b**)

In general, the tree grid that results from applying the adaptation algorithm results in the presence of the aforementioned resolution boundaries and accompanying ghost cells within the domain (see Fig. 2). To define the field values of ghost points, the Basilisk solver uses the downsampling and upsampling operations. The implementation is visually represented for a 1D scenario in Fig. 4. First, downsampling is used to define the field values of ghost points on the high-resolution side of a resolution boundary. Second, an upsampling method is used to define the field values of the ghost points on the coarse side of the resolution boundary. By using this method, the estimation error in the ghost cells’ field values scales with $\zeta$.

The formulations used for downsampling and upsampling as exemplified in Figs. 3 and 4 can be easily extended to two and three dimensions, for so-called quadtree and octree grids, respectively. In order to demonstrate the algorithm and the effect of different $\zeta$ values on the representation of a turbulent field, the next section shows the results of the algorithm applied to a slice of a 3D turbulent field.

The Basilisk solver can run in parallel on many processors by applying a domain decomposition using the Message Passing Interface (MPI). As the grid structure may change during a simulation run, an important issue is load-balancing; the decomposition of the domain between processors must then be modified as the grid is locally refined or coarsened. This is achieved in the Basilisk solver using the natural decomposition of a Z-ordering space-filling curve applied to the quad/octree structure (Griebel and Zumbusch 2001).

**Fig. 5:**
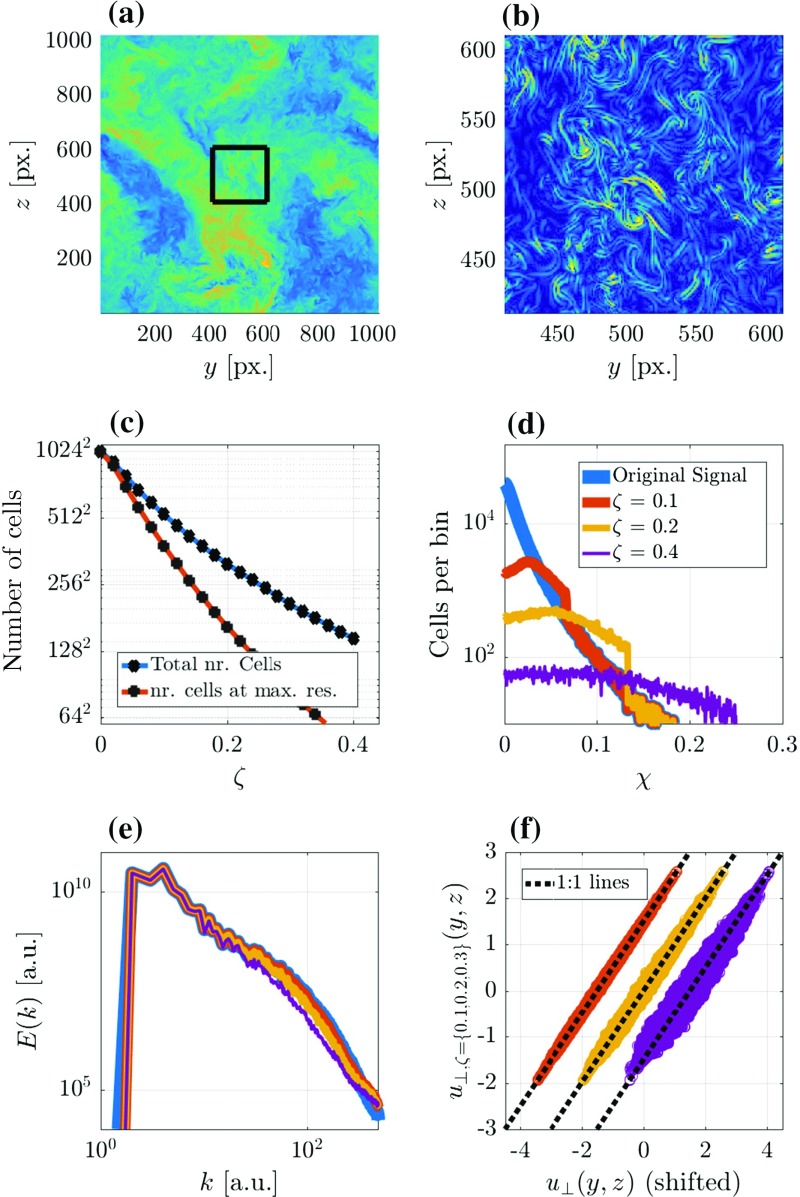
Example of the adaption algorithm applied to a 2D slice of a 3D turbulent field. **a** Shows the data slice of the velocity component in the plane-perpendicular direction ($u_{\perp }$, obtained from Li et al. (2008). **b** Presents the $\chi$ field, evaluated using the method described in Sect. 2.4. Only the centre part of the slice, indicated by the black box in **a**, is shown to reveal the small-scale details in this simulation. **c** shows the grid-cell-number dependence on the chosen refinement criterion ($\zeta$), note the logarithmic vertical axis. A histogram of the $\chi$ field with 512 bins for the original data, and the data corresponding to three $\zeta$ values are presented in **d**. Using the same colour coding as in **d**, power spectra and a direct comparison of the $u_{\perp }(y,z)$ field are shown in **e**, **f**, respectively

### An Example of the Adaptation Algorithm

This section aims to exemplify how the adaption algorithm assesses a discretized signal and adapts the grid according to a refinement criterion $\zeta$. For this purpose, we apply the algorithm to a subset of the data from the simulation of forced isotropic turbulence in Li et al. (2008). The simulation is run using a fixed equidistant grid with $1024^3$ nodes; in terms of the Kolmogorov length scale ($\eta$), the grid spacing ($\varDelta _i$) is $\varDelta _i=2.2 \eta$. For the analysis we assume the data to be resolved well enough, and the results are kindly made available via the Johns Hopkins turbulence databases (http://turbulence.pha.jhu.edu/). We analyze a 2D slice of the data (i.e. $1024^2$ cells) and for simplicity, we only consider the velocity component perpendicular to the sliced plane ($u_{\perp }$). The data are presented in Fig. 5a; using the algorithm described in Sect. 2.1, we can evaluate the $\chi$ field corresponding to the original $u_{\perp }$ field. A section of the resulting field, indicated by the black box in Fig. 5a, is shown in Fig. 5b, where we can clearly see that the estimated discretization error is not distributed uniformly by the equidistant-grid approach that was used in the simulation. Rather, it appears that there are anisotropic structures present, visualized by relatively high $\chi$ values (in yellow). These structures appear to correspond to vortex filaments that characterize the dissipative structures of high-Reynolds-number turbulence (Frisch 1995). This result motivates the application of the grid refinement algorithm to the data sample shown. Note that we cannot add new information by refinement and at this point we do not make any claims regarding what $\chi$ values are reasonable for a turbulence-resolving simulation (this will depend on the numerical formulations and is the topic of a future study). As such, we only allow the algorithm to coarsen the field with a maximum error threshold $\zeta$ (as defined in Eq. 4). The number of grid cells resulting from the application of the adaptation algorithm for a range of $\zeta$ values is shown in Fig. 5c; as expected, the number of grid cells decreases with an increasing $\zeta$ value. Note that the plot also shows that even for the high $\zeta$ values, the grid still contains cells at the maximum resolution.

The main concept of employing the described grid-adaption algorithm is visualized in Fig.5d. Here histograms of the number of grid cells within 512 equally-spaced $\chi$ bins are presented for the original data and the data obtained from applying the grid adaptation technique with three different refinement criteria. It appears that for the original dataset, the histogram is monotonically decreasing with increasing $\chi$. This shows that many grid cells exist where the numerical solution is relatively smooth compared to cells in the tail of the histogram. Hence, if the grid is chosen such that the discretization errors in the latter region do not affect the relevant statistics of the flow evolution, then the grid must be over-refined elsewhere. The histograms of the adapted grids show that the algorithm is able to lower the number of grid cells with low $\chi$ values, such that fewer grid cells are employed. Note that the grid coarsening does not introduce new grid cells with $\chi >2\zeta /3$, as this part of the histogram remains unaltered.

When grid cells with a small but finite $\chi$ value are coarsened, some of the data are lost and in general cannot be exactly reconstructed by interpolation techniques (see Sect. 2.4). In order to assess how the data from the adapted grids compare with the original data, Fig. 5e presents the corresponding power spectra. It appears that none of the adapted grid data are able to exactly reproduce the original power spectrum; more specifically, with increasing $\zeta$ values, the wavenumbers (*k*) that show a significant deviation in *E*(*k*) from the original appear to decrease. We point out that in order to evaluate the spectrum we have linearly interpolated the data from the non-uniform grids to an equidistant grid with $1024 \times 1024$ data points. The choice of the interpolation technique is arbitrary and will pollute the diagnosed spectrum in a non-trivial manner. As such, we directly compare all $1024^2\ u_{\perp }(x,y)$ samples in Fig. 5f, where we see that the deviation of the data from the 1 : 1 line is a function of $\zeta$.

The example presented in Fig. 5 is meant to demonstrate the used adaptation algorithm. The following sections are dedicated to assessing its application to time-dependent numerical simulations of a turbulent field for an atmospheric case.

### Physical Case Set-Up

As indicated in the Introduction, we ran a DNS case from the referenced literature to validate, benchmark and exemplify the adaptive-grid approach. The cases from virtually all atmospheric-turbulence-resolving studies prescribe the periodicity of the solution in the horizontal directions. Unfortunately, at the time of writing, the Basilisk solver cannot yet handle an adaptive grid in combination with periodic boundaries. To circumvent this limitation, we limit ourselves to a case where there is no mean horizontal forcing such that we can apply a no-penetration boundary condition for the normal velocity component at the lateral boundaries. This is supplemented with a Neumann-boundary condition for the tangential velocity components, pressure and buoyancy fields. We realize that this choice might affect the solution and therefore its impact is assessed by re-running the case using a fixed and regular grid with both sets of lateral boundary conditions (not shown). It appears that for the chosen set-up of the case, the simulation results are insensitive to the choice of the horizontal boundary conditions. Note that in future work, we will update the adaptive solver such that periodic boundary conditions can be combined with the AMR technique.

**Fig. 6:**
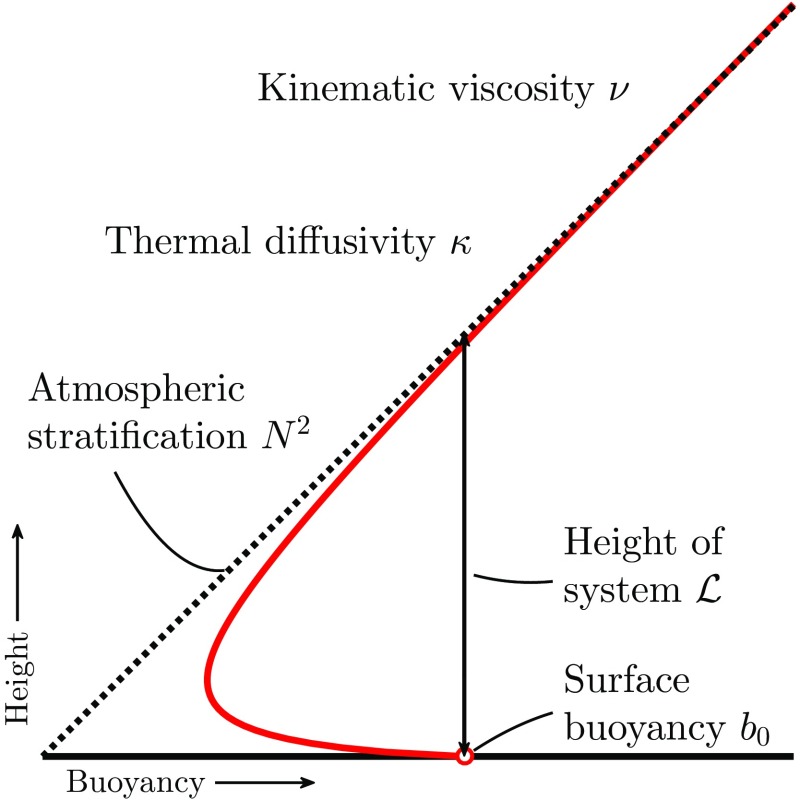
Sketch of the system and its parameters. The red line illustrates a typical buoyancy profile within the CBL during the initial development. Adapted from Heerwaarden and Mellado (2016)

We study a case introduced by Heerwaarden and Mellado (2016) that was designed to investigate the growth and decay of a CBL. In Fig. 6 a schematic overview of the physical system is presented, and in their physical model a linearly stratified fluid at rest with kinematic viscosity ($\nu$) and thermal diffusivity ($\kappa$) is heated from below by a surface with a constant temperature. For generality, buoyancy (*b*) is used as the thermodynamic variable. The buoyancy is related to the potential temperature ($\theta$) according to

5 $$\begin{aligned} b&=\frac{g}{\theta _{\mathrm {ref}}}(\theta -\theta _{\mathrm {ref}}), \end{aligned}$$

where $\theta _{ref}$ is a reference potential temperature and *g* the acceleration due to gravity. The initial linear stratification is expressed as $b(z)=N^2z$, where $N^2$ is the Brunt–Väisälä frequency associated with the initial stratification and *z* is the height above the surface. We assign a surface buoyancy $b_0$ larger than zero. Heerwaarden and Mellado (2016) identified relevant length, time, velocity fluctuation and buoyancy flux scales, $\mathcal {L},T,U$ and *B*, respectively, according to

6a $$\begin{aligned} \mathcal {L}&= \frac{b_0}{N^2}, \end{aligned}$$

6b $$\begin{aligned} T&= \frac{b_0^{2/3}}{N^2\kappa ^{1/3}}, \end{aligned}$$

6c $$\begin{aligned} U&=\frac{b_0^{7/9}\kappa ^{1/9}}{N^{2/3}}, \end{aligned}$$

6d $$\begin{aligned} B&=b_0^{4/3}\kappa ^{1/3}, \end{aligned}$$

and are used to analyze the results in a non-dimensional framework. Two dimensionless groups can be identified that describe the system for any given set of $\{\nu , \kappa , N^2, b_0\}$,

7a $$\begin{aligned} Pr&= \frac{\nu }{\kappa }, \end{aligned}$$

7b $$\begin{aligned} Re&= \left( \frac{b_0^{4/3}}{\nu ^{2/3}N^2}\right) ^{4/3}, \end{aligned}$$

where *Pr* is the Prandtl number and *Re* is the Reynolds number. Note that for $Pr=1$, the definition of the Reynolds number is consistent with $Re={U\mathcal {L}}/{\nu }$.

### Numerical Set-Up and Formulation

For the evolution of the three velocity components ($u_i$), modified pressure (*p*) and buoyancy (*b*), the Navier–Stokes equations for an incompressible fluid are solved under the Boussinesq approximation, according to,

8 $$\begin{aligned} \frac{\partial u_i}{\partial t} +\frac{\partial u_ju_i}{\partial x_j}&= - \frac{\partial p}{\partial x_i} + \nu \frac{\partial ^2 u_i}{\partial x_i^2}+b\delta _{i3}, \end{aligned}$$

9 $$\begin{aligned} \frac{\partial b}{\partial t} + \frac{\partial u_jb}{\partial x_j}&= \kappa \frac{\partial ^2 b}{\partial x_j^2}, \end{aligned}$$

10 $$\begin{aligned} \frac{\partial u_j}{\partial x_j}&= 0, \end{aligned}$$

and with respect to no-slip and a fixed buoyancy ($b_0$) condition at the bottom boundary. At the top boundary, no-penetration with a free-slip condition is used and for the buoyancy, a fixed vertical gradient ($N^2$) is prescribed. Furthermore, a damping layer in the top 25% of the domain is active that damps buoyancy and velocity fluctuations to prevent the artificial reflection of gravity waves at the top boundary. The adaptive-grid runs are initialized with a grid at the minimum resolution that is locally refined to the maximum resolution near the bottom boundary (i.e. $z < \mathcal {L}/10$) before a random perturbation is added to the velocity components and buoyancy field in each grid cell.

Each integration timestep, grid adaptation is based on the estimated error (see Sect. 2.1) of the three velocity components, and the buoyancy field. For each field a refinement criterion ($\zeta$) is specified ($\zeta _{u_{i}},\zeta _b$), where we non-dimensionalize the refinement criteria according to $\xi _b = \zeta _b b_0^{-1}$ and $\xi _{u_{i}}=\zeta _{u_{i}}U^{-1}$. In order to validate the results and assess the performance of the adaptive solver, we iteratively decrease the refinement criterion between runs whilst we limit the minimum grid-box size. This maximum resolution is inspired by Heerwaarden and Mellado (2016), and to limit the degrees of freedom, we choose; $\xi _{u_1}=\xi _{u_2}=\xi _{u_3}= 2.7\times \xi _{b}$. We realize that this choice (based on trial and error) is rather arbitrary, as currently a solid framework of how the refinement criteria should be chosen is still lacking. The results are validated by a comparison with runs using a regular and fixed grid at the maximum resolution, performed with the Basilisk and MicroHH flow solvers: MicroHH is the numerical code used by Heerwaarden and Mellado (2016) to obtain their results. This code represents a state-of-the-art flow solver that is dedicated to studying atmospheric systems (Heerwaarden and Mellado 2016; Shapiro et al. 2016); for a detailed description of the MicroHH code see Heerwaarden et al. (2017). In addition, the fixed grid results of the Basilisk and MicroHH flow solvers are compared to each other.

We choose $Pr = 1$ and $Re = 3000$ with a domain size of $3\mathcal {L} \times 3\mathcal {L} \times 3\mathcal {L}$ and simulate the evolution of the system until the physical time $t = 45T$. In order to limit the computational costs, the evolution of the Basilisk-based run with a fixed regular grid is only computed until $t = 10T$. To illustrate the physical size of such a numerical experiment in reality; for a domain size of $0.5\ \mathrm {m} \times 0.5\ \mathrm {m} \times 0.5\ \mathrm {m}$ and $\theta _{\mathrm {ref}} = 21\ ^{\circ }\mathrm {C}$, the corresponding parameters are: $\mathcal {L} = 0.16\ \mathrm {m}$, $\theta _{\mathrm {bottom}} = 36\ ^{\circ }\mathrm {C}$ and $T = 153\ \mathrm {s}$. This could be interpreted as a modest laboratory experiment.

The simulations are performed with Surfsara’s supercomputer Cartesius located in Amsterdam, The Netherlands (SURE 2017). An overview of the different runs, including the number of cores used, integration timesteps and total run time is listed in Table 1. Additional information on the case set-up for both models can be found at:

**Table 1:** Overview of the different simulation run details. In the top section a reference name, the used solver, grid type, the (maximal) numerical grid resolution, lateral boundary conditions and refinement criterion ($\xi _b$, if applicable) are listed for each run

Run name	Code	Grid	$n_x \times n_y \times n_z$ (Maximal)	Lateral BCs	$\xi _b$
MicroHH	MicroHH	Fixed and stretched	$512^2\times 387$	Periodic	–
BA-512$^3$	Basilisk	Fixed	$512^3$	Neumann and No-pen.	–
BA-0.0025	Basilisk	AMR	$512^3$	Neumann and No-pen.	0.0025
BA-0.005	Basilisk	AMR	$512^3$	Neumann and No-pen.	0.005
BA-0.01	Basilisk	AMR	$512^3$	Neumann and No-pen.	0.01

Run name	Number of cores	Integration steps at $t/T=\{10,\ 45\}$	Total wall clock time (D:HH:MM)		
MicroHH	64	{13920, 35670}	0:12:22		
BA-512$^3$	64	{14073, *(35670)*} *(estimated)*	2:16:12 ($t/T=10$)		
BA-0.0025	96	{14095, 30144}	2:10:30		
BA-0.005	96	{14061, 28704}	1:18:19		
BA-0.01	96	{14167, 25544}	1:02:16		

In the bottom section the used number of cores, the total amount of integration steps taken at $t/T =\{10, 45\}$ and the total wall clock time of each run are presented Italic values indicate the estimated values

Basilisk: http://basilisk.fr/sandbox/Antoonvh/freeconv.c^1^

MicroHH: https://github.com/microhh/microhh/tree/master/cases/vanheerwaarden2016

## Results

### Grid Structure

**Fig. 7:**
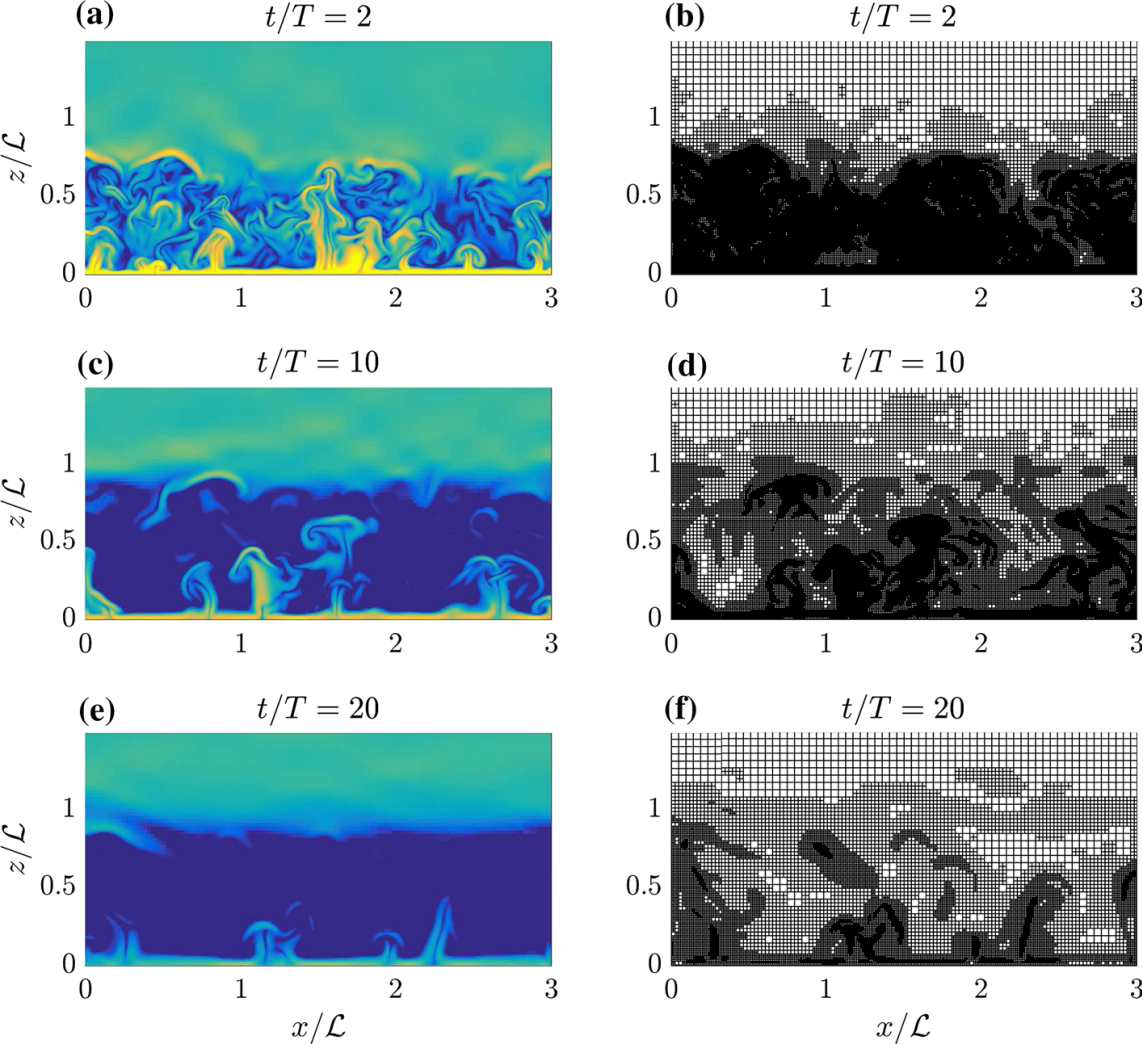
Vertical slices of the $\Vert \nabla b\Vert$ field (left column) and the corresponding numerical grid (right column) in the lowest half of the domain. The top, middle and bottom rows represent snapshots taken at $t/T = \{2,10,20\}$, respectively. These snapshots are taken from the adaptive-grid run BA-0.0025

First, we study the evolution of the solution and grid structure qualitatively. Vertical slices of the magnitude of the gradient of the buoyancy field ($\Vert {\nabla b}\Vert$) and the used grid at $t = \{2, 10, 20\}T$ for run BA-0.0025 are presented in Fig.  7. At $t = 2T$ a complex grid structure is generated by the AMR algorithm, and within the ABL the grid is refined at locations where vigorous turbulent structures are present. Above the ABL (i.e. $z/\mathcal {L} > 1$), turbulence is absent and the grid is coarse. Both effects are appealing from a physical perspective as the computations are focused on the regions where the activity is present. As the physical time progresses, the boundary layer becomes more neutrally stratified and the turbulence intensity decreases. And again, in response, the adaptive-grid algorithm has coarsened the grid at $t = 10T$. This remarkable effect is even more pronounced at $t = 20T$, where the coarsened regions have grown in size, indicating that the number of grid cells decreases over time. Physically speaking, this is facilitated by the fact that the size of the smallest eddies increases as turbulence decays.

**Fig. 8:**
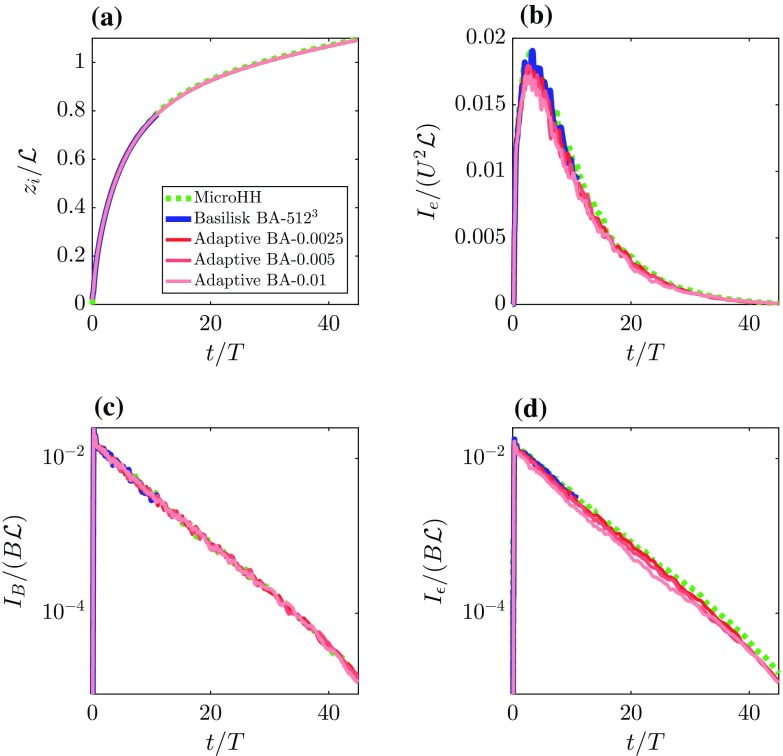
Time series of the domain integrated quantities, **a** boundary-layer height $(z_i)$, **b** kinetic energy ($I_e$), **c** buoyancy flux ($I_B$) and **d** dissipation rate ($I_\epsilon$) according to Eq. 11. The results are obtained with both Basilisk and MicroHH solvers using fixed grids and Basilisk using the adaptive mesh refinement algorithm. Note that plots **c**, **d** use a logarithmic scale

### Validation

Next we compare the results obtained with the AMR and fixed-uniform-grid runs. Following Heerwaarden and Mellado (2016), we compare the domain integrated quantities: a boundary-layer height $z_i$ that is based on the buoyancy profile, kinetic energy $I_\mathrm{e}$, buoyancy flux $I_\mathrm{b}$ and dissipation $I_\epsilon$ according to,

11 $$\begin{aligned} z_i&= \frac{2}{N^2}\int ^{\infty }(\langle {b}\rangle -N^2z) \mathrm {d}z, \end{aligned}$$

12 $$\begin{aligned} I_\alpha&=\int ^{\infty } \langle \alpha \rangle \mathrm {d}z, \end{aligned}$$

where $\alpha$ is a dummy variable for $\{e, b,\epsilon \}$ and $\langle \alpha \rangle$ denotes the horizontally-averaged value of the quantity $\alpha$. Figure 8a shows the evolution of the boundary-layer height, where good agreement between all simulations is found. The boundary-layer height is an integral measure of the amount of buoyancy (i.e. analogous to heat) in the system, though due to the case set-up, this integral quantity is not a very sensitive measure to assess the accuracy of the resolved turbulent motions. Therefore, we focus on higher-order statistics. In general, the evolution of the total kinetic energy shows similar behaviour between all runs (see Fig. 8). Nevertheless small discrepancies on the order of 5% are present, particularly between the runs with the adaptive grid and the fixed uniform grids, and as expected, this discrepancy decreases when the refinement criterion is more strict. In order to analyze the evolution of kinetic energy in further detail, Fig. 8c presents the evolution of the domain-integrated buoyancy flux, which represents the energy-production rate for this system. The buoyancy flux agrees well for all different runs and the observed differences between the runs are a result of turbulent fluctuations within the chaotic system rather than systematic discrepancies. This indicates that the overall structure and characteristics of the energy-producing motions are resolved accurately for all runs, and for free convection, these motions are associated with the large thermal plumes. In order to assess the representation of the small-scale structures in these simulations, Fig. 8d presents the evolution of the resolved energy-dissipation rate. Compared to the fixed-grid runs, the AMR-based runs slightly underestimate the resolved absolute dissipation, an aspect that is present throughout the simulation. Again, the discrepancy appears to be controlled by the refinement criterion, for which using stricter (i.e. smaller) criteria the results seem to converge towards the values found with the fixed-grid runs. The fact that the runs diagnosed with a lower dissipation rate are also associated with lower kinetic energy indicates that a small part of the dissipation has a numerical/non-physical origin.

**Fig. 9:**
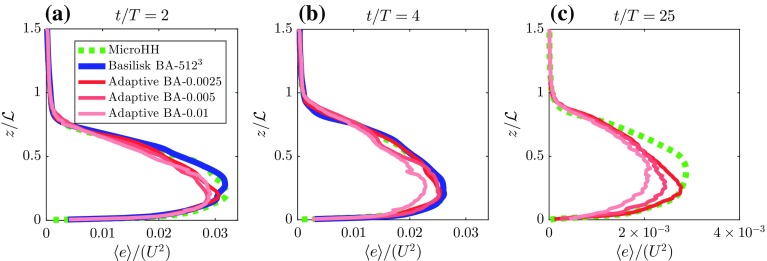
Vertical profiles of the horizontally-averaged kinetic energy ($\langle e \rangle$) at $t/T = \{2,4,25\}$ in left, middle and right plot, respectively. The results are obtained with both Basilisk and MicroHH solvers using fixed grids and Basilisk using the adaptive mesh refinement algorithm. Note that in panel **c** the horizontal axis is rescaled and that regular-grid computations with the Basilisk solver are not available (see text, Sect. 2.4)

Figure 9 shows the vertical profiles of the kinetic energy at $t/T = \{2, 4, 25\}$, and shows discrepancies at $t/T=2$ between all runs. The highly chaotic flow structure at this early stage of the simulation could explain some of the differences. However, consistent with Fig. 8b, the adaptive-grid runs show a systematically lower kinetic energy content over the entire domain. At $t/T = 4$, the profiles of the fixed-grid runs agree well, and furthermore, the energy found in the adaptive-grid run BA-0.0025 also compares well. It can be seen from the time series in Fig. 8b that for $t/T < 5$, the evolution of kinetic energy shows large fluctuations. Therefore, we also compare the energy profiles at $t/T=25$, where we see again that the fixed-grid run still contains more energy than the adaptive-grid runs. Again, the adaptive run with the smallest refinement criterion is closest to the fixed-grid profile compared to the other adaptive-grid runs.

Although it appears that the adaptive-grid algorithm is able to refine the grid at locations of the turbulent structures, discrepancies in the simulation results remain present. This can be explained by the fact that the process of refining and coarsening the mesh relies on a linear interpolation strategy for defining values on new grid cells. This interpolation introduces additional errors compared to a simulation that employs a static grid, and these errors are similar to the truncation errors of fixed-grid advection schemes and thus lead to similar additional numerical dissipation of energy. More accurate interpolation techniques could be tested to limit the error due to interpolation; therefore, this relevant aspect will be studied in more detail in the future.

**Fig. 10:**
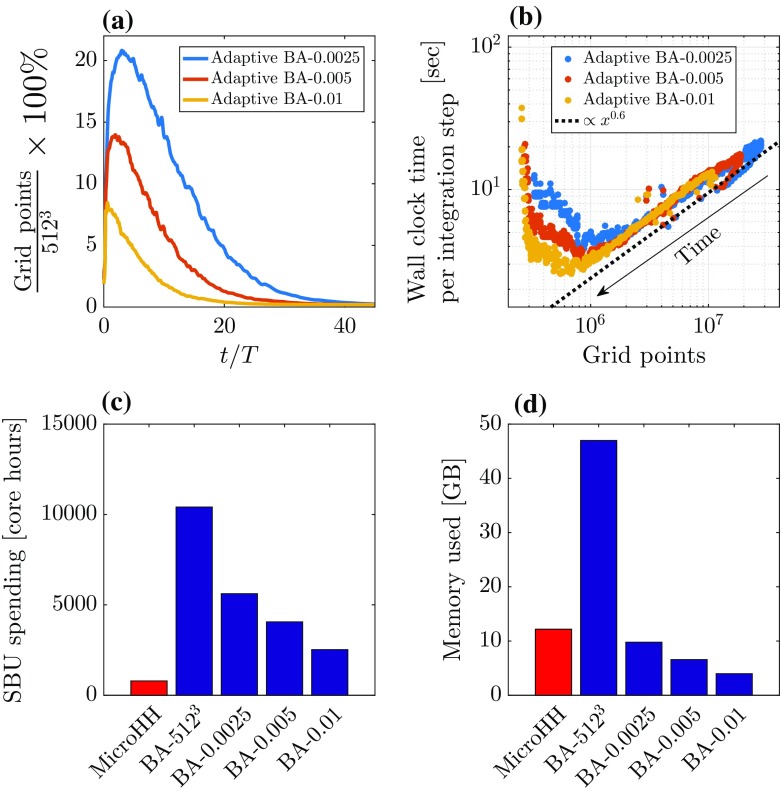
Overview of the performance characteristics of the adaptive and fixed-grid simulation runs. **a** Time series of the number of grid points for the adaptive runs normalized by the maximum-resolution value (i.e. $512^3$). **b** Scatter plot of the wall clock time per integration step versus the used number of grid cells in the adaptive-grid runs. **c** The total amount of system billing units (SBU, i.e. $number\,of\,cores \times hours$) spending on each simulation run. Note that the value for BA-512$^3$ is estimated as if it were run until $t/T = 45$. **d** The total RAM memory used in each simulation run in gigabytes (GB)

### Performance

As discussed in the introduction, for highly dynamic flow configurations such as a diurnal cycle, model performance may benefit from the AMR approach. Although the present case of decaying convection is less dynamic than a full diurnal cycle, it is tempting to compare the simulation performance of the AMR-based run to its counterparts using a fixed and regular grid. Thereupon, several performance characteristics are presented in Fig. 10. Figure 10a shows, for the AMR-based runs, the evolution of the number of grid cells that appear to be controlled by the refinement criterion, in which a smaller value causes the algorithm to use a more refined grid. As illustrated in the snapshots of Fig. 7, the number of grid cells varies significantly over the course of the simulation. Supposedly, the computational resources are distributed more efficiently over time. Furthermore, even in the run with the strictest refinement criterion, the number of grid cells does not exceed 21% of the maximum-resolution value. Figure 10b shows how the computational speed (i.e. defined here as wall clock time per integration timestep) is correlated with the number of grid cells. It appears that there are several regimes in which the performance is affected by the number of grid cells. For a large number of grid cells (i.e. $> 10^6$) the amount of integration timesteps per second increases with a decreasing number of grid cells, indicating that the solver does indeed speed up when the grid is coarsened. Note that the simulations apply many grid cells in the early stage of the runs i.e. at the right-hand side of Fig. 10b and uses fewer cells as time progresses (towards the left-hand side of Fig. 10b). However, as denoted by the $x^{0.6}$-scaling line, in this regime the simulation speed is not linearly dependent on the amount of grid cells. Furthermore, for lower number of grid points (i.e. $< 10^6$) the simulation speed appears to decrease when the simulation runs with fewer grid cells, i.e. there is a performance penalty for coarser grids! Possible causes for these performance characteristics are listed below:

1. For this case, the grid structure of the coarsened grids at later stages in the simulation contains a relatively larger fraction of resolution boundaries (see Fig. 7). These boundaries are associated with additional overhead as they require special attention by the solver (see Sect. 2.1).
2. The number of used processors (linked to domain decomposition for parallelization) is fixed throughout the simulations. Therefore, the relative overhead of MPI-domain communication routines compared to actual calculations increases as the number of grid cells decreases.
3. For coarse grids, the physical timestep taken per integration timestep increases (Courant–Friedrichs–Lewy criterion). Diagnostic analysis of the solution is performed with a regular interval in the physical time, i.e. $\varDelta t = T$ for profiles and slices and $\varDelta t = T/20$ for the domain-integrated quantities. The frequency of calls to diagnostic routines increases (i.e. say, calls per 100 integration steps) on average resulting in an increased effort per integration step.

In Fig. 10c the amount of system billing units (i.e. the used $number\,of\,cores \times hours$) spending for the different runs is presented. Before an interpretation of the results is made, it is important to realize that the performance of a simulation run is a function of many aspects that ranges from the details of the hardware configuration to the exact case set-up. Therefore, the results presented here are intended as an illustration rather than as absolute values. Nevertheless, it is clear that the MicroHH run is notably less computationally demanding compared to the runs performed with the Basilisk solver. This can be explained by the different numerical schemes that are employed. Most notably, for obtaining the pressure field, the Basilisk code uses a multigrid strategy for solving the corresponding Poisson equation whereas the isotropic-fixed grid in MicroHH facilitates the usage of a spectral Poisson solver. Although the spectral method requires more MPI communication for parallelization when using a large number of processors, it is known to be more efficient (Fornberg 1998). If we compare the adaptive and non-adaptive simulation runs performed with the Basilisk solver, we do see a considerable decrease in computational costs for the adaptive method runs.

In Fig. 10d the memory used for the different simulation runs is presented, and compared to the fixed-grid runs, the adaptive-grid runs require less memory. This is due to the fact that the maximum number of grid cells is considerably lower than the number of grid cells in the fixed-grid runs (see Fig.  10a). From this perspective, the adaptive-grid approach is also attractive for applications where the available memory is limited. However, even though the run with MicroHH employs many more grid cells, the required memory is comparable to that of run BA-0.0025, meaning that per grid cell, the MicroHH code is more efficient in terms of memory.

## Outlook: Towards Adaptive Mesh Refinement in Atmospheric LES

**Fig. 11:**
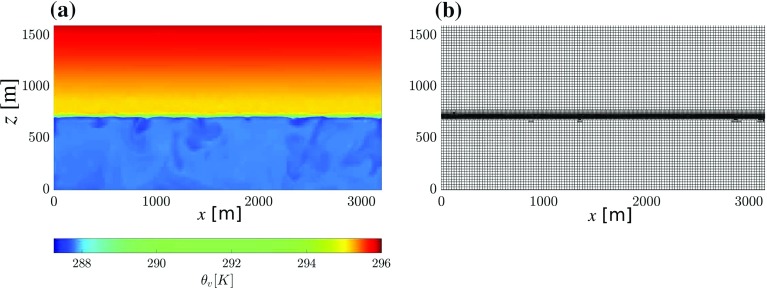
Snapshots of **a** the vertical slices of the virtual potential temperature field, and **b** the numerical grid at $$t = 3\ $$h. The case is based on Bretherton et al. (1999)

We have based our test and performance benchmark on an idealized flow configuration of a CBL using DNS, providing a ground truth for our intercomparison. In the future, we plan to study more practically-oriented cases by using an LES formulation. For many atmospheric cases, LES is preferred over DNS, because it provides an efficient tool for studying high-Reynolds-number flows. Therefore, the next step is to test the AMR approach in combination with an LES formulation. In this section, we briefly discuss some preliminary results on this topic. Because this is part of ongoing research, we do not perform a quantitative discussion of the test runs, the results and performance characteristics. The presented results aim to exemplify the AMR method for a different case and show the flexibility of the AMR approach. The example is based on the LES intercomparison study case by Bretherton et al. (1999), in which a boundary layer is filled with a smoke cloud that cools from the top due to longwave emission. The boundary layer is initially capped by a strong temperature inversion (i.e. 7 $\mathrm {K}$ over 50 m) at $z \approx 700\ \mathrm {m}$ and rises over the course of the simulation due to entrainment. The inversion layer is identified as a region where turbulent length scales are suppressed and turbulent motions are anisotropic due to the stable stratification. As such, this region requires a high resolution to capture the predominant turbulent structures accurately. In constrast, the convective turbulence in the boundary layer itself can be captured on a relatively coarse grid (Sullivan and Patton 2011). Accordingly, we decided not to base the grid adaptation upon the estimated discretization error in the representation of the velocity-component fields, but only on the estimated error in the smoke-cloud fraction and temperature fields. With such an approach the AMR algorithm does not refine the mesh in order to resolve the small turbulent structures in the near-neutral boundary layer, but allows the LES to employ the SGS model effectively in this region. In this run, the numerical grid varies by three levels of refinement, i.e. between 25 and 3.125 m. Figure 11 presents snapshots of the temperature and numerical grid taken at $t = 3$ h after initialization. It is clear from Fig. 11a that an inversion layer is present, while Fig. 11b shows that the numerical grid has a high resolution in the region of the inversion layer and remains coarse in the boundary layer itself. Furthermore, we see the subsiding shells in the boundary layer that are qualitatively similar to those observed in the laboratory experiment performed by Jonker and Jiménez (2014).

For this case, the AMR algorithm dynamically adapts to the flow by redirecting the grid refinement to those regions of the spatial domain where it is required. Hence in this case, adaptation is predominantly spatially focussed, whereas in the DNS case the refinement was most prominent in the temporal domain (see Fig. 10a). As such, both examples in this study are complementary and both effects (spatial and temporal adaptive grid refinement) are expected to play an important role in future simulations of full diurnal cycles (cf. Fig. 1).

Finally, we note the following; the present cases where restricted to spatially homogeneous set-ups, where ‘scale separation’ naturally occurs through the internal variability of turbulence, originating from the non-linearity of the governing equations. In reality, heterogeneity in the *surface boundary conditions* also becomes important and provides an additional cause of scale separation that may call for adaptive grid refinement. For example, refinement may be preferred at sharp transitions between different types of land use, such as the land–sea interface.

## Discussion and Conclusions

We have introduced and tested an adaptive mesh refinement (AMR) method for studies of the atmospheric boundary layer (ABL). This work is motivated by a desire to numerically study highly dynamic cases. Such cases are characterized by a high degree of scale separation throughout the spatial and temporal domain. This work should be viewed as the first step in our AMR-based research that assesses the usage of an AMR method for studies of the ABL. We have based our adaptive-grid simulations on the flow solver implemented in the Basilisk code.

The method is tested using DNS based on a case introduced by Heerwaarden and Mellado (2016), describing the growth and subsequent decay of a CBL. The AMR algorithm was able to identify the time-varying turbulent regions in the domain and refined/coarsened the grid accordingly. The AMR-based simulations can reproduce the simulation results of their fixed grid counterparts with minor discrepancies. Furthermore, the AMR algorithm can be tuned to apply more grid cells such that these discrepancies are suppressed. For all AMR runs, the number of grid cells varies significantly over time, resulting in more efficient simulations compared to using a regular fixed grid with identical numerical formulations. This provides a proof of principle for the AMR method regarding ABL systems.

For this case, a numerical solver dedicated to ABL systems (MicroHH) outperformed all other runs in terms of computational efficiency, indicating that there is an overhead associated with the use of the adaptive solver. In general, the exact impact of this overhead depends on the details of the studied case. The most challenging ABL systems typically owe their complexity to the dynamical interplay between various processes at different length and time scales. Hence, the AMR technique is likely to be more favourable as complexity increases. More specifically, as discussed in Popinet (2011), the cost of an adaptive simulation, relative to a constant resolution simulation (*G*) is expected to scale as

13 $$\begin{aligned} G=\frac{C_a\varDelta ^{-D}}{C_c\varDelta ^{-3}} = \frac{C_a}{C_c}\varDelta ^{3-D}, \end{aligned}$$

where $C_a$ and $C_c$ are constants related to the absolute speed of the computation for the adaptive- and constant-resolution simulations, respectively ; $\varDelta$ is the ratio of the minimum to the maximum scale of the physical system (i.e. a measure of scale separation) and *D* is the effective (or fractal) dimension of the physical process (which is necessarily $\le 3$). In the present study, $\varDelta$ is relatively large (i.e of order $10^{-2}$) and the computational gain using the adaptive method is correspondingly small, whereas for challenging cases $\varDelta$ can be several orders of magnitude smaller, with a correspondingly larger potential gain in efficiency of the adaptive method relative to constant-resolution methods. This important aspect of the overall scaling behaviour is illustrated in “Appendix 1” for a canonical flow set-up. The results shown herein thus motivate our continued research using the AMR technique.

## Figures and Tables

**Fig. 12:**
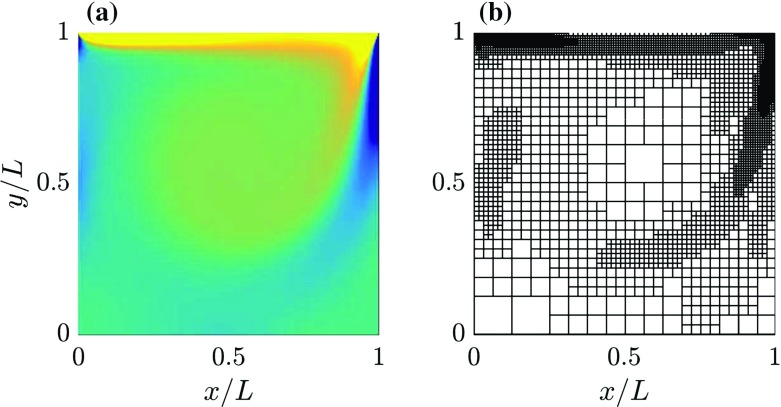
Snapshots of **a** the vorticity field, and **b** the numerical grid at $t = t_{end}$ for the lid-driven cavity simulation with $Re_{lid}=500$

**Fig. 13:**
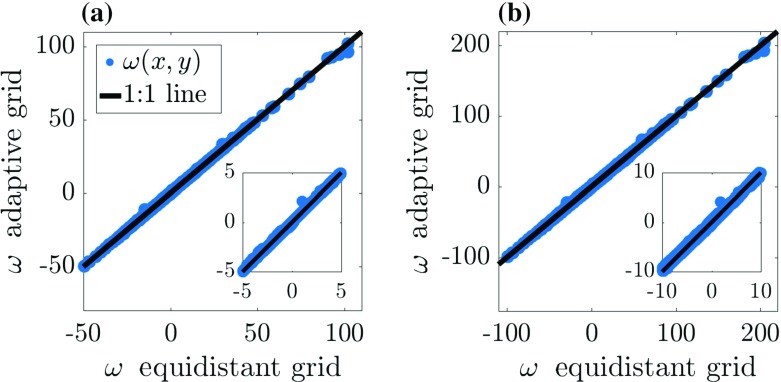
Validation of the vorticity field ($\omega$) from the adaptive grid simulation against the results obtained with a fixed equidistant grid. For **a** $Re_{lid}=250$, and **b** $Re_{lid}=500$. The inserts show a zoom-in (i.e. rescaled axes), containing $\approx \,95\%$ of the total number of data points

**Fig. 14:**
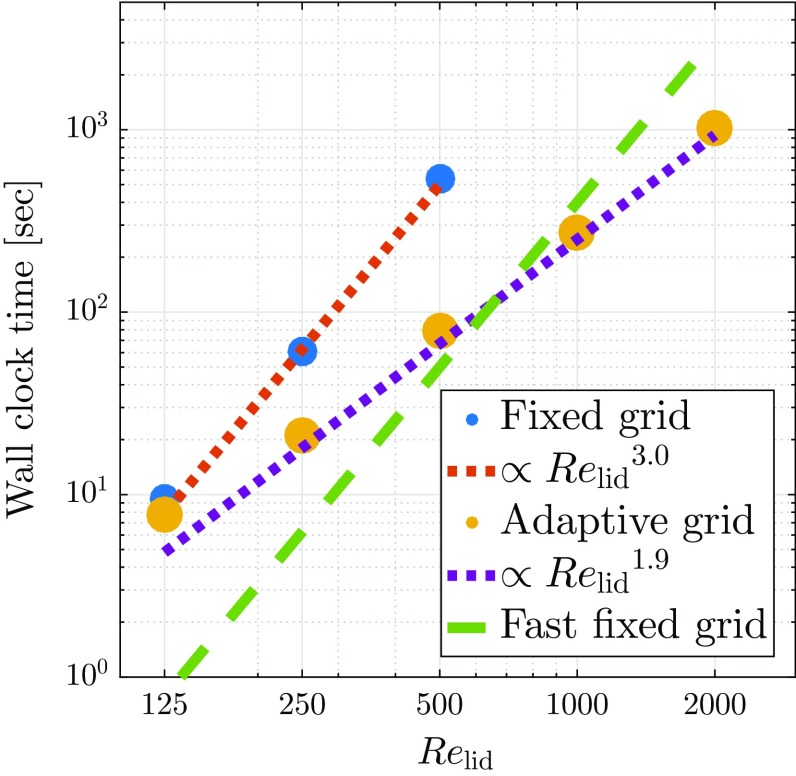
The correlation of the computational costs and the Reynolds number ($Re_{lid}$) for different approaches. The green line represents the theorized results from a solver that is an order of magnitude faster than the fixed-grid approach that we have used (blue dots)

## Appendix 1: The Lid-Driven Cavity in Two Dimensions

We study the relation between the computational costs and the scale separation for a simulation of a fluid in a lid-driven cavity in two dimensions, and compare the results from a regular fixed grid and the adaptive-grid-refinement approach as is presented herein. The chosen physical set-up consists of a no-slip box (size $L \times L$), in which an incompressible fluid with kinematic viscosity $\nu$ is set in motion by the top lid that moves with a constant velocity ($U_{lid}$) in the left-to-right direction. It is well known that this configuration results in a large circulation cell within the domain. With system parameters $L, \nu$ and $U_{lid}$ we can identify a Reynolds number ($Re_{{lid}}$) according to

14 $$\begin{aligned} Re_{{lid}}=\frac{U_{lid}L}{\nu }. \end{aligned}$$

In order to study the effect of varying scale separation on the performance statistics, the simulations cover a range of different Reynolds numbers. Following the analysis of Clercx and Heijst (2017) on vortex-wall interactions in two dimensions, we take the (minimum) grid-box size inversely proportional to the Reynolds number. As such, the Reynolds number represents the separation of scales in our simulations (i.e. $\varDelta$ in Eq. 13). The runs are initialized with the fluid at rest and the flow evolution is simulated for a physical time $t_{end}=20 L/U_{lid}$. For the adaptive grid simulations, a refinement criterion for the velocity components $\zeta =0.005U_{{lid}}$ is chosen. All runs are performed using a single processor core. A snapshot of the vorticity field and the corresponding grid structure at $t=t_{end}$ for $Re_{{lid}} = 500$ are presented in Fig. 12. The maximum resolution of this simulation corresponds to a $256 \times 256$ grid. First, the solution is validated against the results obtained with the fixed equidistant-grid runs in Fig. 13. Here the vorticity fields ($\omega (x,y)$) obtained from the fixed-grid and adaptive-grid simulations are directly compared for the runs with $Re_{{lid}}=\{250, 500\}$. We conclude that the chosen refinement criterion in sufficiently small to accurately reproduce the results obtained with the equidistant grid. Second, Fig. 14 presents the scaling of the computational costs with the Reynolds number. The simulation costs when employing the fixed and equidistant grid appear to scale with the third power of the Reynolds number. This exponent can be understood from the fact that the total number of grid cells scales with the Reynolds number to the second power (i.e. in 2D, doubling the resolution requires four times as many grid cells). Furthermore, the well-known numerical stability criterion of Courant–Friedrichs–Lewy limits the timestep and scales inversely proportional to the grid-box size, meaning that the total number of timesteps is proportional to the Reynolds number. Combined, the computational costs scale with the Reynolds number to the power of $(2\mathrm {\ space}+1\mathrm {\ time}=)3$. This analysis holds for all equidistant-grid approaches, and as such, we can anticipate the computational costs when using an equidistant-grid code that is an order of magnitude faster than the solver we have chosen for our fixed-grid approach (i.e. the Basilisk solver running in fixed-grid mode). Interestingly, with an increasing Reynolds number, the observed scaling of the adaptive grid simulations is favourable compared to the equidistant grid counterpart. The observed scaling (i.e $\propto Re^{1.9}$) reflects that the resolution requirement is not space filling. Although that for the lower Reynolds numbers (i.e. $Re \lesssim 1000$), the (theorized) fast equidistant-grid solver is more efficient than the adaptive grid approach, there exists a crossing point where the latter technique becomes the more effective option. This feature is indicative to all processes in nature. in which, with an increasing scale separation, the space-filling approach of an equidistant grid represents the worst-case scenario, neglecting the so-called fractal dimension of the problem. Note that this concept also applies to three-dimensional turbulence (see Chap. 8 in Frisch 1995). However, the corresponding scaling behaviour for ABL cases is not obvious.

## Footnotes

1 From a users’ perspective, the case definition for the adaptive-grid runs is (subjectively) not more involved than the fixed-grid counterpart. The more complex adaptation-specific formulations are addressed by a low-level part of the Basilisk toolbox that does not require explicit attention from the users’ side.
